# A Novel, Non-Apoptotic Role for Scythe/BAT3: A Functional Switch between the Pro- and Anti-Proliferative Roles of p21 during the Cell Cycle

**DOI:** 10.1371/journal.pone.0038085

**Published:** 2012-06-27

**Authors:** Sheila T. Yong, Xiao-Fan Wang

**Affiliations:** Department of Pharmacology and Cancer Biology, Duke University Medical Center, Durham, North Carolina, United States of America; Universidade Federal do Rio de Janeiro, Brazil

## Abstract

**Background:**

Scythe/BAT3 is a member of the BAG protein family whose role in apoptosis has been extensively studied. However, since the developmental defects observed in *Bat3*-null mouse embryos cannot be explained solely by defects in apoptosis, we investigated whether BAT3 is also involved in cell-cycle progression.

**Methods/Principal Findings:**

Using a stable-inducible *Bat3*-knockdown cellular system, we demonstrated that reduced BAT3 protein level causes a delay in both G1/S transition and G2/M progression. Concurrent with these changes in cell-cycle progression, we observed a reduction in the turnover and phosphorylation of the CDK inhibitor p21, which is best known as an inhibitor of DNA replication; however, phosphorylated p21 has also been shown to promote G2/M progression. Our findings indicate that in *Bat3*-knockdown cells, p21 continues to be synthesized during cell-cycle phases that do not normally require p21, resulting in p21 protein accumulation and a subsequent delay in cell-cycle progression. Finally, we showed that BAT3 co-localizes with p21 during the cell cycle and is required for the translocation of p21 from the cytoplasm to the nucleus during the G1/S transition and G2/M progression.

**Conclusion:**

Our study reveals a novel, non-apoptotic role for BAT3 in cell-cycle regulation. By maintaining a low p21 protein level during the G1/S transition, BAT3 counteracts the inhibitory effect of p21 on DNA replication and thus enables the cells to progress from G1 to S phase. Conversely, during G2/M progression, BAT3 facilitates p21 phosphorylation by cyclin A/Cdk2, an event required for G2/M progression. BAT3 modulates these pro- and anti-proliferative roles of p21 at least in part by regulating cyclin A abundance, as well as p21 translocation between the cytoplasm and the nucleus to ensure that it functions in the appropriate intracellular compartment during each phase of the cell cycle.

## Introduction

BAT3 (also known as BAG-6 or Scythe) is a member of the Bcl-2 associated anthanogene (BAG) family of proteins. BAG proteins are thought to function as molecular bridges between the Hsp70 (heat shock protein) molecular chaperones and their target proteins [1], [2]. BAT3 was first discovered as a member of a group of genes located within the class III region of the human major histocompatibility complex on chromosome 6, and has been extensively studied for its role in regulating apoptosis under various stress conditions such as DNA damage and endoplasmic reticulum-related stress [3], [4], [5]. BAT3 has been shown to be required for p53 acetylation, which is critical for the enhancement of p53 transcriptional activity in response to DNA damage [4]. In addition, BAT3 has recently been demonstrated to play a critical role in regulating a number of biochemical processes, such as facilitating the proteasomal degradation of the apoptosis inducing factor and Hsp70-2/HspA2 [3], [5], [6], [7], although the mechanisms appear to be diverse and remain to be elucidated. Interestingly, the phenotype of *Bat3*-null mice also demonstrates the importance of the biological function of BAT3. *Bat3*-null mice suffer from pre-natal lethality with severe defects in kidney, lung and neural development due primarily to the loss of proper control in the balance of apoptosis and proliferation [8]. These findings suggest that BAT3 might also be involved in cell-cycle regulation.

The eukaryotic cell division cycle is regulated in large part by cyclin/cyclin-dependent kinase (CDK) complexes, which are in turn modulated by CDK inhibitors (CKIs) such as p21^WAF1/Cip1^ (referred to as p21 hereafter) that bind to specific cyclin/CDK complexes [9], [10]. p21, a member of the Cip/Kip family of CKIs, regulates cell-cycle progression by inhibiting DNA replication [11], [12]. In response to DNA damage, p21 activates the G1 cell-cycle checkpoint and appears to steer cells away from apoptosis and toward arrest or even senescence [12], [13], [14], [15], [16]. Many p21 studies have focused on how the oscillation of p21 protein level affects the cell cycle, and the general consensus is that p21 has to be degraded to enable cell-cycle progression [17], [18], [19], [20], [21]. Paradoxically, p21 has also been shown to promote cell-cycle progression during G1 when it enhances cyclin D/Cdk4 assembly and regulates the intracellular localization of this complex [22]. In addition, p21 has recently been demonstrated to enhance cyclin B/Cdk1 activity during G2/M progression [23]. However, how the two opposing roles of p21 are regulated during the cell cycle is largely unknown.

In this study, we report evidence for a functional link between BAT3 and p21 during specific phases of the cell cycle. We show that during the G1/S transition, BAT3 negatively regulates p21 protein abundance to allow smooth progression through this boundary. In contrast, during G2/M progression, BAT3 promotes p21 phosphorylation by cyclin A/Cdk2; this phosphorylation has previously been shown to be required for cyclin B/Cdk1 activation [23]. Moreover, p21 and BAT3 appear to be co-localized in a cell-cycle-dependent manner, and BAT3 appears to be required for the proper translocation of p21 between the cytoplasm and the nucleus. Additionally, we observed that BAT3 regulates Cdk2 kinase activity through cyclin A abundance. Therefore, our findings define a new role for BAT3 as a functional switch for modulating the pro- and anti-proliferative roles of p21 in cell-cycle regulation, as well as to ensure the proper intracellular localization of p21 during the cell cycle.

## Results

### Bat3-knockdown (KD) Cells Show Defects in both G2/M Progression and G1/S Transition

**Figure 1:**
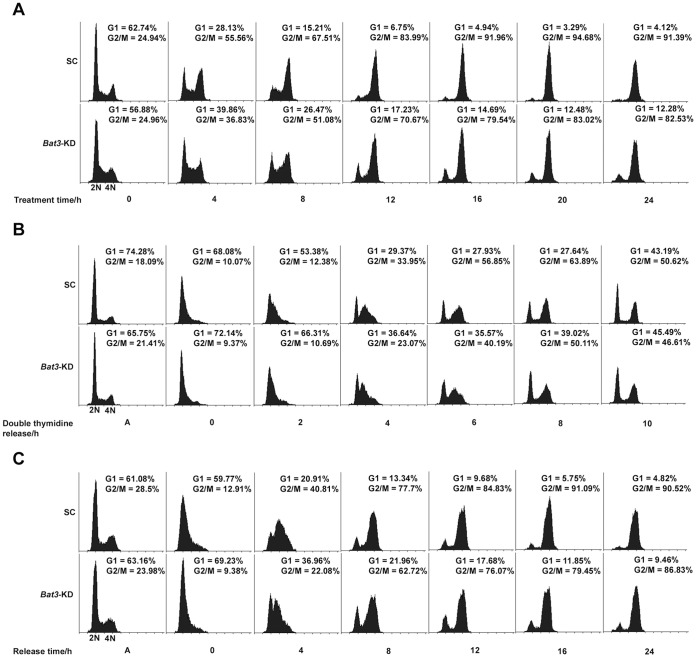
*Bat3* knockdown leads to defects in G2/M progression and G1/S transition. DNA profiles of scrambled control (SC) and *Bat3*-knockdown (*Bat3*-KD) U2OS stable cell lines following (A) treatment with 100 ng/ml nocodazole, (B) release from G1/S synchronization, and (C) release from G1/S synchronization into medium with 100 ng/ml nocodazole. Cell count is shown on the vertical axis while DNA content is shown on the horizontal axis. The proportions of cells in G1 (*2N* DNA) and G2/M (*4N* DNA) of the cell cycle were quantified and are indicated for each time point.

For this study, we used a stable, inducible human osteosarcoma U2OS cell line-based *Bat3* knockdown system previously generated in our lab [24] which allows us to reduce BAT3 protein level following doxycycline (Dox) treatment. To determine the effects of *Bat3* knockdown on cell-cycle progression, U2OS cells stably integrated with either a scrambled control (SC) or *Bat3*-specific shRNA construct were cultured in Dox-containing medium and subsequently treated with nocodazole. These cells were harvested every 4 h following nocodazole treatment over a 24-h period and their DNA profiles were analyzed by flow cytometry (Figure 1A). We found that the *Bat3*-KD cells exhibited a delayed accumulation in G2/M and decreased efficiency progressing through G1 compared to the SC cells. To ensure that the *Bat3*-knockdown-induced cell-cycle phenotype is not simply due to nocodazole itself, we synchronized the U2OS stable cell lines at G1/S using double thymidine block and analyzed their DNA profiles following release. As shown in Figure 1B, similar results were obtained as we observed that a larger proportion of *Bat3*-KD cells remained in G1 throughout the 10-h time course.

To confirm this finding, we synchronized the SC and *Bat3*-KD U2OS cells at G1/S and released them into medium containing nocodazole. Our results show that *Bat3-*KD cells indeed progressed more slowly from G1 into S phase, as indicated by the consistently larger G1 population at all time points examined (Figure 1C).

These results thus far suggest that BAT3 promotes cell-cycle progression by facilitating both the G1/S transition and G2/M progression, since a decrease in BAT3 protein level causes a lag at both of these points during the cell cycle. Based on these findings, we proceeded to investigate the functional link between BAT3 and regulatory components of the cell cycle.

### Bat3 Knockdown Leads to Reduced p21 Phosphorylation in Nocodazole-arrested Cells

**Figure 2:**
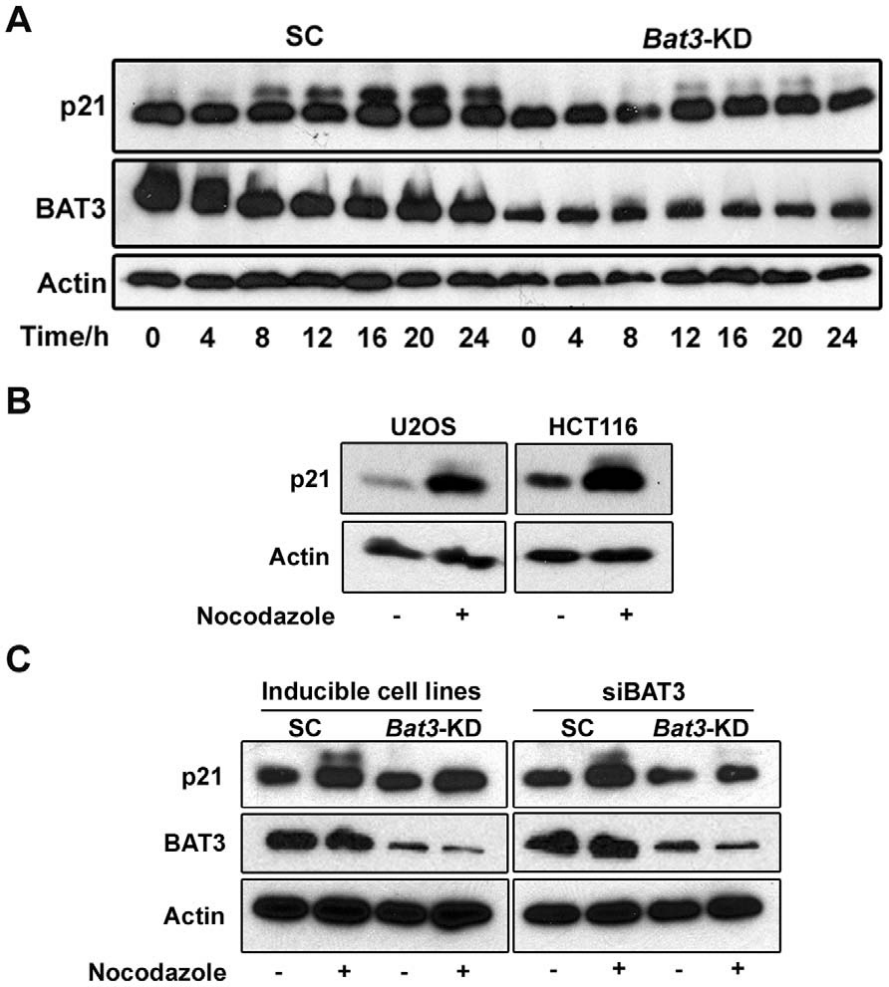
*Bat3* knockdown reduces the extent of p21 phosphorylation following nocodazole treatment. (A) Western blot for p21 using lysates from scrambled control (SC) and *Bat3*-knockdown (*Bat3*-KD) U2OS stable cell lines treated with 100 ng/ml nocodazole. The slower migrating band corresponds to the phosphorylated form of p21. (B) Western blot for p21 for parental U2OS and HCT116 cells treated with nocodazole as previously described [23]. (C) Western blot for p21 for parental U2OS cells treated with 800 ng/ml nocodazole for 24 h. siRNA was used to transiently reduce *Bat3* expression (right panel). Lysates from the stable, inducible U2OS cell lines were used as a positive control (left panel).

To further explore the role of BAT3 in regulating cell-cycle progression, we first focused on the G2/M phase. A recent study by Dash and El-Deiry found that cells lacking p21 experience a G2/M delay when treated with nocodazole. They proposed that p21 phosphorylation is required to activate cyclin B/Cdk1 activity, which is in turn required for G2/M progression [23]. As shown in Figure 2A, p21 in lysates prepared from nocodazole-treated SC cells migrated as two distinct bands during SDS-PAGE, and that the intensity of the slower-migrating band was reduced in the *Bat3*-KD cells. It has been previously observed that phosphorylated p21 migrates more slowly during SDS-PAGE [23], [25], [26]. Hence, to verify that the slower migrating band is due to the phosphorylated form of p21, we incubated the lysate from SC cells treated with nocodazole for 24 h with λ-phosphatase. In accordance with previous studies, we observed a reduction in the abundance of the slower migrating form of p21 following *in vitro* dephosphorylation (Figure S1). Therefore, our results not only agree with those of Dash and El-Deiry [23] but also suggest that BAT3 is responsible for facilitating p21 phosphorylation. Additionally, we observed that *in vitro* dephosphorylation of phosphorylated p21 was inhibited by the presence of the phosphatase inhibitors sodium fluoride and sodium orthovanadate (Figure S1, lanes 3 and 4). Interestingly, we observed that diluting the lysate with the phosphatase assay buffer containing only protease inhibitors could also collapse the two bands into one (Figure S1, first lane). This observation suggests the presence of phosphatase(s) in the lysate that dephosphorylated p21 when the phosphatase inhibitors present in the lysate were diluted below their effective concentrations.

To verify that the effect of *Bat3* knockdown on p21 phosphorylation that we observed is not cell-type specific, we treated both parental U2OS cells and human colon cancer HCT116 cells with nocodazole as previously described [23] and subsequently prepared cell lysates for western blot analysis. Consistent with earlier findings [23], we observed that p21 was also phosphorylated in these cells following nocodazole treatment (Figure 2B). Additionally, we performed a transient knockdown of *Bat3* in the parental U2OS cells using siRNA and observed that p21 hyperphosphorylation following nocodazole treatment was also reduced (Figure 2C). Finally, to control for the usage of Dox in these experiments, we treated *Bat3*-KD cells with nocodazole both in the presence and absence of Dox. We found that in the absence of Dox (i.e. when BAT3 protein level was not reduced), p21 was phosphorylated in the *Bat3*-KD cells to a similar extent as in the SC cells (Figure S2). Taken together, these observations suggest that BAT3 facilitates p21 phosphorylation in nocodazole-treated cells and subsequently promotes G2/M progression.

As BAT3 has been proposed to regulate apoptosis [27], [28], [29], we wanted to determine whether the treatment of the U2OS cells with nocodazole results in apoptosis as measured by caspase-3 cleavage. Since the role of BAT3 in apoptosis regulation has been reported to depend on activated caspase-3 [29], [30], the presence of cleaved caspase-3 in the lysates would indicate that the cells were undergoing apoptosis as a result of nocodazole treatment. As shown in Figure S3, we did not detect caspase-3 cleavage during western blot analysis of lysates prepared from both SC and *Bat3*-KD cells following nocodazole treatment. This observation suggests that these cells do not undergo apoptosis under our experimental conditions. Therefore, there is no evidence that the apoptotic function of BAT3 contributes to the cell-cycle phenotype we have observed.

### Bat3-KD Cells are Less Efficient at Resuming Cell-cycle Progression Upon Release from Nocodazole Arrest and Show Reduced Oscillation in p21 Protein Level

**Figure 3:**
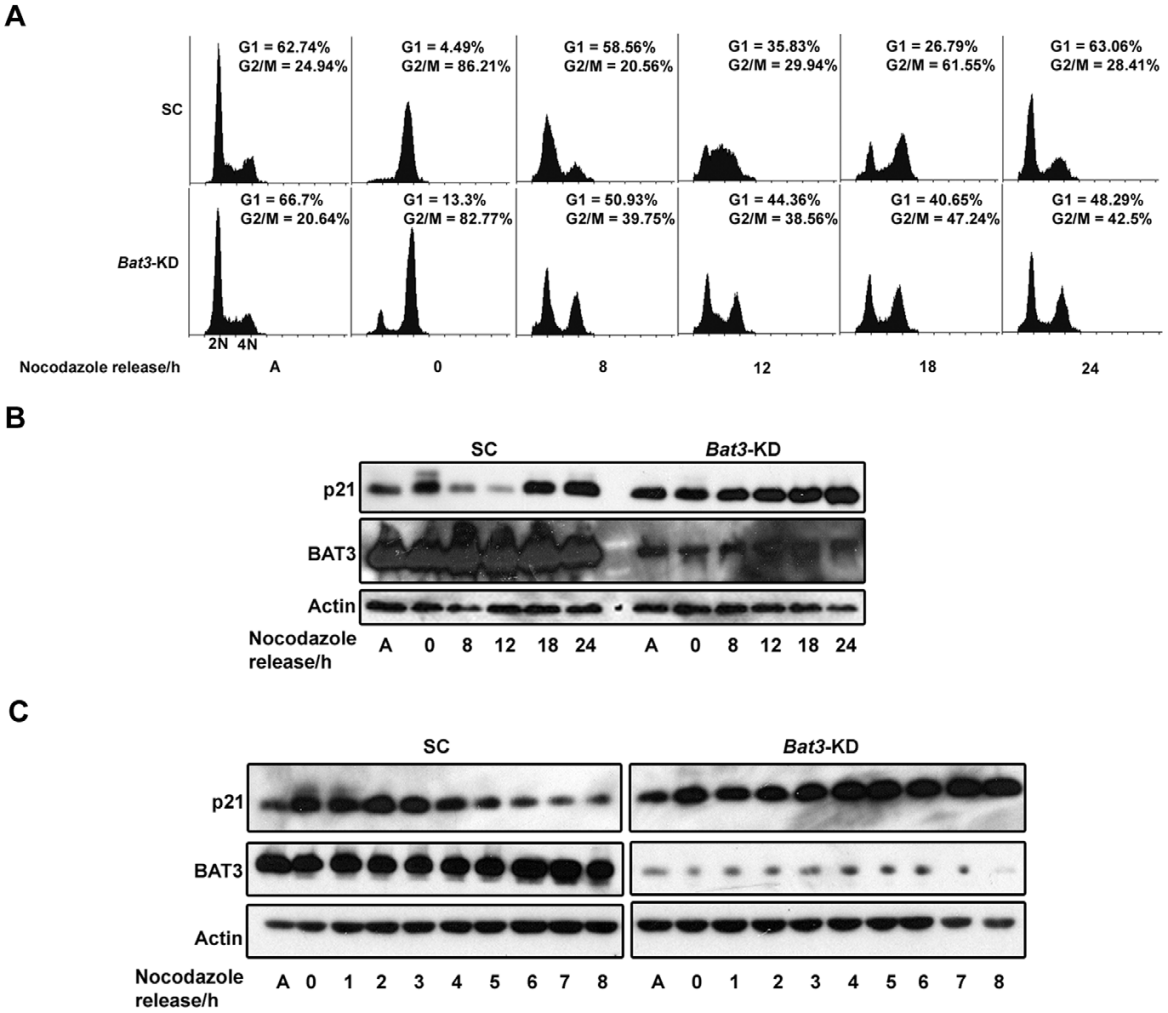
*Bat3* knockdown results in reduced efficiency of cell-cycle resumption and negatively affects the oscillation of p21 protein level in cells released from nocodazole arrest. (A) Flow cytometry analysis of scrambled control (SC) and *Bat3*-knockdown (*Bat3*-KD) U2OS cells arrested with 100 ng/ml nocodazole for 24 h and subsequently released into full culture medium. Cells were harvested at the indicated time points. Cell count is shown on the vertical axis while DNA content is shown on the horizontal axis. The proportions of cells in G1 (*2N* DNA) and G2/M (*4N* DNA) of the cell cycle were quantified and are indicated for each time point. (B) and (C) Western blot for p21 using lysates from SC and *Bat3*-KD U2OS cells arrested with 100 ng/ml nocodazole for 24 h and subsequently released into full culture medium. The slower migrating band corresponds to the phosphorylated form of p21. A, asynchronous population.

Since *Bat3*-KD cells accumulate more slowly in G2/M following nocodazole arrest, we wanted to determine whether the ability of these cells to recover from nocodazole arrest is also affected. We arrested SC and *Bat3*-KD cells with nocodazole for 24 h and determined their DNA profiles over a 24-h period following release. While the SC cells successfully resumed the cell cycle and progressed through the various cell-cycle phases, the *Bat3*-KD cells were less efficient at doing so (Figure 3A). As expected, the *Bat3*-KD cells accumulated more slowly in G2/M following nocodazole arrest. Interestingly, a proportion of these cells that managed to progress into G2/M were less efficient at resuming cell-cycle progression upon release, resulting in a relatively static population even after release. Similarly, a proportion of cells that managed to progress into G1 upon release remained in G1, once again leading to a relatively stationary population. These results reveal that in addition to regulating the progression of cells into G2/M, BAT3 also regulates the progression from G1 into S phase. In light of these findings, we also investigated the role of BAT3 in regulating the G1/S transition and describe those results later in this report.

Given that BAT3 is required for cells to efficiently resume the cell cycle upon release from nocodazole arrest, we wanted to determine whether BAT3 also regulates p21 protein level in these cells. The level of p21 protein has been shown to oscillate throughout the cell cycle and to have a major impact on cell-cycle progression [17], [20]. To do this, we performed western blotting for p21 using lysates prepared from the SC and *Bat3*-KD cells that had been released from nocodazole arrest. In the SC cells, p21 protein level decreased at 8 h, was at its lowest at 12 h, and increased again by 18 h following release; however, this oscillation was not detected in the *Bat3*-KD cells (Figure 3B). The oscillation in p21 protein level we observed for the SC cells is consistent with previous findings [17] and also agrees with their DNA profiles, which show that these cells were transitioning from G1 to S phase at 8 h and progressing through S phase at 12 h after release (Figure 3A). On the other hand, since the G1 and G2/M populations for the *Bat3*-KD cells remained relatively constant following release, the level of p21 protein in these cells did not oscillate but instead continued to increase. To analyze this phenomenon in greater detail, we performed the same experiment but harvested cells every hour up until 8 h following release instead. As shown in Figure 3C, p21 phosphorylation and total p21 protein level in the SC cells began to diminish 1 h and 4 h after release, respectively. On the other hand, p21 phosphorylation was much less evident in the *Bat3*-KD cells while p21 protein abundance increased throughout the 8-h period following release.

**Figure 4:**
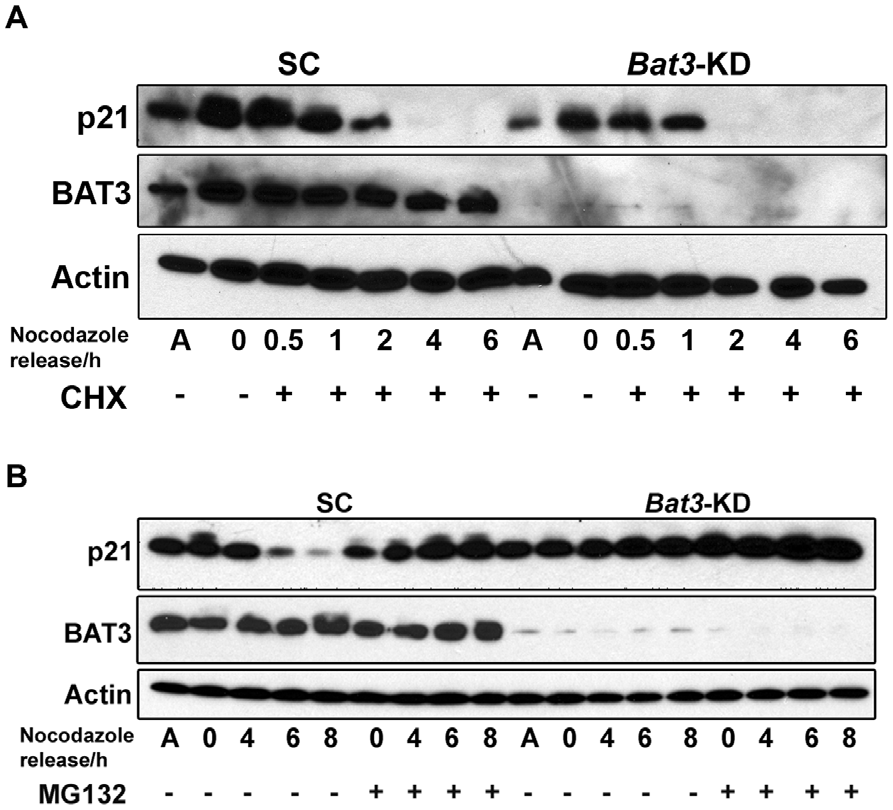
Hyperphosphorylation and abundance of p21 protein in the U2OS stable cell lines upon release from nocodazole arrest into CHX and MG132. (A) Western blot for p21 for lysates from scrambled control (SC) and *Bat3*-knockdown (*Bat3*-KD) U2OS cells arrested with 100 ng/ml nocodazole for 24 h and subsequently released into full culture medium containing 40 µg/ml CHX. (B) Western blot for p21 for lysates from SC and *Bat3*-KD U2OS cells arrested with 100 ng/ml nocodazole for 24 h and subsequently released into full culture media either with or without 20 µM MG132. For the MG132 samples, cells were pre-treated with 20 µM MG132 for 2 h before release. A, asynchronous population; CHX, cycloheximide.

Since we observed that p21 protein level oscillates in cells that have been released from nocodazole arrest, we wanted to examine the relationship between p21 hyperphosphorylation leading up to G2/M progression and the oscillation in p21 protein level following release from nocodazole arrest. The SC and *Bat3*-KD cells were arrested in nocodazole for 24 h and then released into medium containing cycloheximide (CHX) to inhibit protein synthesis. The phosphorylation of p21 in these cells was subsequently determined by western blotting (Figure 4A). We observed that in the SC cells, the slower-migrating band corresponding to the phosphorylated form of p21 started to decrease at 1 h and became undetectable by 2 h after release whereas the faster-migrating, hypophosphorylated form of p21 was still detectable at that time (Figure 4A, lanes 4 and 5, respectively). However, this phenomenon was less evident in the *Bat3*-KD cells since the starting level of phosphorylated p21 was lower, and total p21 protein became undetectable in these cells 2 h after release (Figure 4A, lane 12). Our findings suggest that the accumulation of p21 protein in the *Bat3*-KD cells was maintained by continuous p21 synthesis. A dynamic equilibrium may exist between p21 synthesis and degradation that is tightly regulated during the cell cycle. This equilibrium would shift in favor of either the synthesis or degradation of p21 depending on the cell-cycle phase. When BAT3 protein level is reduced, the equilibrium appears to be shifted in favor of p21 synthesis (Figures 3C and 4A). Hence, the drastic decline in p21 protein level observed in the *Bat3*-KD cells in the presence of CHX suggests that these cells attempted to restore the equilibrium by increasing p21 degradation. Taken together, these results suggest BAT3 promotes p21 phosphorylation during G2/M progression, which in turn allows p21 to be degraded as the cells resume cell-cycle progression following release from nocodazole-induced arrest.

To further demonstrate the importance of p21 phosphorylation during G2/M progression, we again arrested the SC and *Bat3*-KD cells with nocodazole but released them into medium containing MG132 to inhibit the 20 S proteasome. Since the degradation of mitotic components was inhibited in these cells, we predicted that the phosphorylated form of p21 would continue to be present. As shown in Figure 4B, treating the SC cells with MG132 preserved phosphorylated p21, but this effect was not observed in the *Bat3*-KD cells. These observations agree with our hypothesis that BAT3 is required for p21 phosphorylation during G2/M progression, and suggest that this phosphorylation event triggers the subsequent decline in p21 protein abundance, allowing the cells to exit mitosis and enter the subsequent cell cycle.

Our results thus far show that phosphorylated p21 is unstable in cells released from nocodazole arrest (thus exiting mitosis). However, it remains to be determined whether phosphorylation of p21 leads to its degradation or if it is dephosphorylated prior to its degradation, although there is preliminary evidence suggesting the latter (see the Potential Mechanism and Discussion sections). Nevertheless, our findings highlight the importance of BAT3 in regulating p21 protein level and function during G2/M progression.

### BAT3 Regulates the G1/S Cell-cycle Transition by Regulating p21 Protein Abundance

We observed earlier that the *Bat3*-KD cells progressed from G1 to S phase more slowly compared to the SC cells, indicating that more *Bat3*-KD cells remained in G1 after release from G1/S synchronization by double thymidine block (Figure 1B and 1C). These observations suggest that in addition to regulating G2/M progression, BAT3 may also regulate the G1/S transition during the cell cycle.

**Figure 5:**
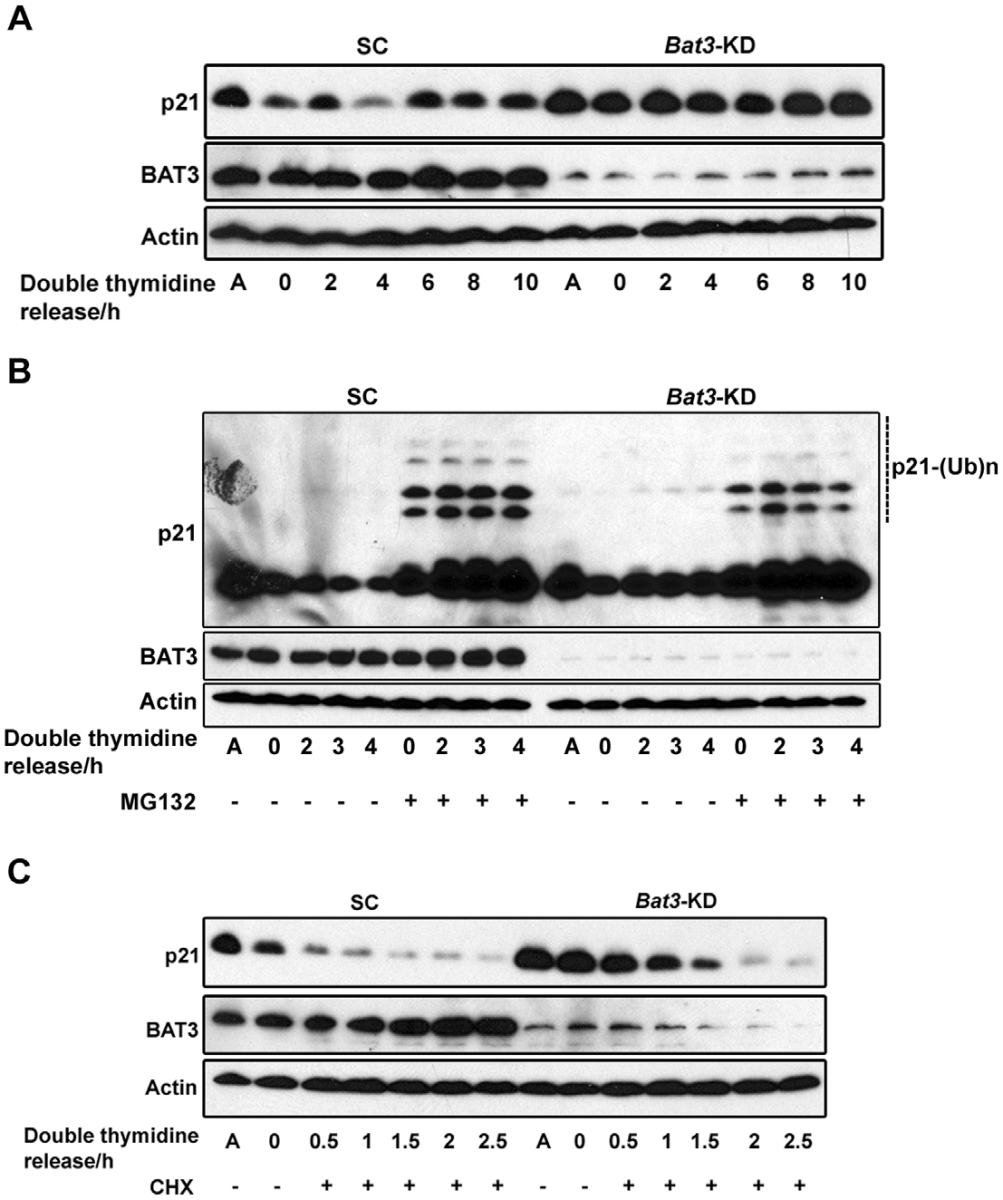
*Bat3* knockdown causes a defect in the oscillation of p21 protein level in G1/S-synchronized cells. (A) Western blot for p21 for scrambled control (SC) and *Bat3*-knockdown (*Bat3*-KD) U2OS cells following release from G1/S synchronization. (B) Western blot for p21 for G1/S-synchronized SC and *Bat3*-KD U2OS cells that were released into full culture media either with or without 20 µM MG132. For the MG132 samples, cells were pre-treated with 20 µM MG132 for 2 h before release. (C) Western blot for p21 for G1/S-synchronized SC and *Bat3*-KD U2OS cells that were released into full culture medium containing 40 µg/ml CHX. A, asynchronous population; CHX, cycloheximide.

Since p21 has been demonstrated to inhibit DNA synthesis [31], [32], [33], we wanted to determine whether the G1/S delay we observed with the *Bat3*-KD cells was due to changes in p21 regulation during G1 and S phase of the cell cycle. We performed western blotting for p21 using lysates from the U2OS stable cell lines synchronized at G1/S and harvested every 2 h over a period of 10 h after release. Following release from double thymidine block, the level of p21 protein in the SC cells oscillated throughout the cell cycle; however, this oscillation was less pronounced in the *Bat3*-KD cells (Figure 5A). We also obtained similar results with cells that were synchronized at G1/S and released into medium containing nocodazole (Figure S4). These results suggest that the G1/S delay observed for the *Bat3*-KD cells might be due to an excess of p21 when the cells attempt to enter S phase. Another important observation is that in the *Bat3*-KD cells released from G1/S synchronization into medium containing nocodazole, p21 phosphorylation was reduced (Figure S4, lanes 12–14). This observation is consistent with our results concerning p21 phosphorylation following nocodazole treatment described above.

We proposed above that a dynamic equilibrium exists between p21 synthesis and degradation that is tightly regulated during cell-cycle progression. In this regard, we observed that this equilibrium was shifted toward p21 synthesis in the *Bat3*-KD cells released from nocodazole arrest. Therefore, we wanted to determine whether this equilibrium is also disrupted during the G1/S transition in the *Bat3*-KD cells. We first examined whether the lack of observed oscillation in p21 protein level in *Bat3*-KD cells released from G1/S synchronization is due to a defect in p21 degradation. p21, which is degraded during S phase, has been shown to be an *in vitro* target of the SCF^Skp2^ ubiquitin ligase [20], [34]; however, p21 degradation *in vivo* during the G1/S transition has also been reported to be ubiquitination-independent [35], [36]. Therefore, we wanted to determine if p21 ubiquitination is altered in *Bat3*-KD cells. The SC and *Bat3*-KD U2OS cells were synchronized at G1/S and subsequently released into medium containing MG132. As shown in Figure 5B, although both SC and *Bat3*-KD cells accumulated ubiquitinated forms of p21, the levels of ubiquinitated p21 were slightly lower in the *Bat3*-KD cells. This observation suggests that the ubiquitination of p21 begins as the cells enter S phase, reducing the level of p21 protein *in vivo* and thus allowing efficient S phase entry. However, since the difference observed between the levels of ubiquitinated p21 in the SC and *Bat3-KD* cells is slight, BAT3 does not appear to play a major role in promoting p21 ubiquitination during the G1/S transition.

Next, we examined whether the half-life of p21 is affected in *Bat3*-KD cells released from G1/S synchronization. G1/S-synchronized SC and *Bat3*-KD cells were released into medium containing CHX and the p21 protein level in these cells was determined by western blotting. We found that *Bat3*-KD cells expressed a higher basal level of p21, which could also contribute to the G1/S delay observed for these cells (Figure 5C). Since p21 was degraded in both SC and *Bat3*-KD cells as they progressed through S phase, a greater abundance of p21 protein in the *Bat3*-KD cells suggests that these cells need more time to degrade p21 before entering S phase, resulting in the observed delay. Interestingly, we also observed a drastic decline in p21 protein level in the *Bat3*-KD cells at 1.5 h following release (Figure 5C, lanes 12 and 13). These findings indicate that the accumulation of p21 protein in the *Bat3*-KD cells during the G1/S transition was also maintained by continuous p21 synthesis. Thus, the dynamic equilibrium between p21 synthesis and degradation was also shifted towards p21 synthesis. An increase in p21 synthesis would result in a higher level of p21 protein in the *Bat3*-KD cells, causing a G1/S delay. Taken together, our results indicate that BAT3 facilitates the G1/S transition by regulating the oscillation in p21 protein level.

### Potential Mechanism for BAT3-mediated Regulation of p21 During the Cell Cycle

#### The role of p53 and Cdk2

**Figure 6:**
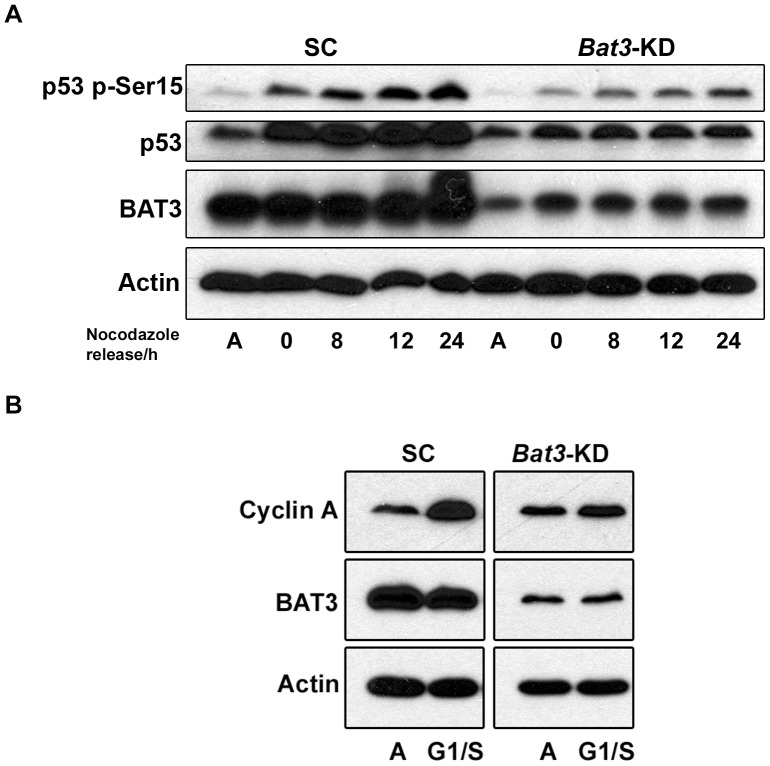
The potential mechanism of BAT3-mediated p21 regulation during the cell cycle. (A) Western blot for phosphorylated p53 at Ser15 and total p53 protein for scrambled control (SC) and *Bat3*-knockdown (*Bat3*-KD) U2OS cells following release from G2/M synchronization. (B) Western blot for cyclin A for SC and *Bat3*-KD U2OS cells synchronized at G1/S. A, asynchronous population.

Both p53-dependent and independent pathways have been implicated in regulating p21 protein expression [11], [37]. Therefore, we wanted to determine the effects of *Bat3* knockdown on p53 using our U2OS stable cell lines. We determined whether the p53 protein abundance is altered in the G2/M-synchronized *Bat3-KD* cells by performing western blotting for p53. Our results show that both the levels of p53 phosphorylation at Ser15 and total p53 protein were lower in the *Bat3*-KD cells than in the SC cells (Figure 6A). Since p53 phosphorylation at Ser15 has been shown to increase p53 stability and enhance its transcriptional activity [38], [39], our observations suggest that the higher p21 protein level in the *Bat3*-KD cells is unlikely to be due to increased p53 transcriptional activity. In fact, knocking down *Bat3* expression appears to negatively affect both the phosphorylation of Ser15 and total p53 protein abundance. Hence, it is likely that BAT3 regulates p21 in a p53-independent manner during cell-cycle progression. Our analysis of p21 mRNA level in *Bat3*-KD cells released from G2/M synchronization by semi-quantitative RT-PCR also revealed that the p21 mRNA level in the *Bat3*-KD cells remained relatively constant at all the time points examined during the 24-h period following release (Figure S5A), suggesting that the upregulation of p21 transcription is unlikely to contribute to the accumulation of p21 protein in these cells. *Bat3* knockdown has also been shown to result in a decrease in p300-mediated p53 acetylation and the subsequent reduction of its transcriptional activity [4]. Taken together, these observations suggest that the accumulation of p21 protein we observed in the *Bat3*-KD cells is unlikely to be dependent on p53.

We confirmed the p21 phosphorylation observed in nocodazole-treated SC cells in our study by performing a λ-phosphatase treatment assay (Figure S1). Additionally, we also observed that diluting the lysate with phosphatase reaction buffer containing only protease inhibitors, without added phosphatase or phosphatase inhibitors, could also collapse the two bands into one. This observation suggests the presence of a phosphatase or phosphatases(s) in the lysate that dephosphorylated p21 when the phosphatase inhibitors present in the lysate were diluted to below their effective concentrations (Figure S1, first lane). These observations as well as previously reported findings suggest that p21 phosphorylation initiates a chain of events resulting in G2/M progression, p21 dephosphorylation and its subsequent degradation (see Discussion).

Previous studies have suggested that cyclin A/Cdk2 is the kinase responsible for phosphorylating p21 during G2/M progression. For instance, two forms of p21 with different mobility shifts have been shown to form a complex with cyclin A in mouse embryonic fibroblasts arrested at G2/M [25]. Cyclin A has also been reported to co-localize with p21 in nocodazole-treated cells, and the inhibition of Cdk2 kinase activity resulted in decreased p21 phosphorylation in G2/M-arrested cells [23]. Therefore, we wanted to investigate whether BAT3 plays a role in regulating Cdk2 activity. We observed that Cdk2 activity is not inhibited by phosphorylation at Tyr15 in *Bat3*-KD cells arrested in mitosis, suggesting that the regulation of this kinase by BAT3 is likely to be indirect (Figure S5B). Moreover, we determined that cyclin A is less abundant in *Bat3*-KD cells synchronized at either G1/S or G2/M (Figure 6B, data not shown). These results suggest that Cdk2 kinase activity is reduced in the *Bat3*-KD cells due to lower cyclin A abundance.

### Functional Interaction between BAT3 and p21 and their Co-localization During the Cell Cycle

The intracellular localization of p21 has previously been reported to be important for its regulation and activity. For instance, the nuclear localization of p21 is required for its phosphorylation [23] and has also been proposed to facilitate G2/M progression [25]. Furthermore, the degradation of p21 at the G1/S border has been reported to occur solely in the nucleus [35], [36], [40], [41]. Therefore, we wanted to determine whether the localization of BAT3 is cell-cycle-dependent, and if it has any effect on the localization of p21.

**Figure 7:**
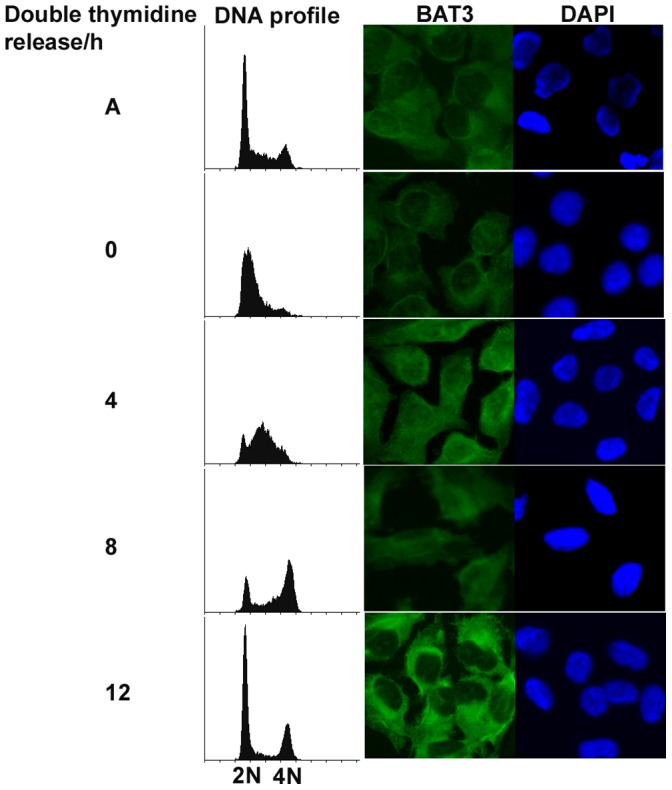
BAT3 localization is cell-cycle-dependent. Parental U2OS cells were harvested every 4 h following release from G1/S synchronization, fixed and stained for BAT3. A, asynchronous population.

First, we synchronized parental U2OS cells at the G1/S boundary and collected cells every 4 h after release for anti-BAT3 immunostaining. As shown in Figure 7, BAT3 was found in the nuclei of some cells and in the cytoplasm of others in an asynchronous population (top panel). In G1/S-synchronized cells, BAT3 was predominantly seen in the perinuclear region (0 h). As the cells progressed into S and G2/M phases, BAT3 was translocated into the nuclei (4 h and 8 h, respectively). Finally, when the cells returned to G1, BAT3 was almost completely excluded from their nuclei (12 h). This observation indicates that BAT3 localization is dynamic and may be subject to cell-cycle regulation. In particular, BAT3 translocates from the cytoplasm to the nucleus of cells as they progress through the cell cycle and back into the cytoplasm as the cells proceed into the next cell cycle. Cell-cycle-dependent BAT3 localization was also observed in SaOS-2 and CCD-34Lu cells (Figures S6A and S6B).

**Figure 8:**
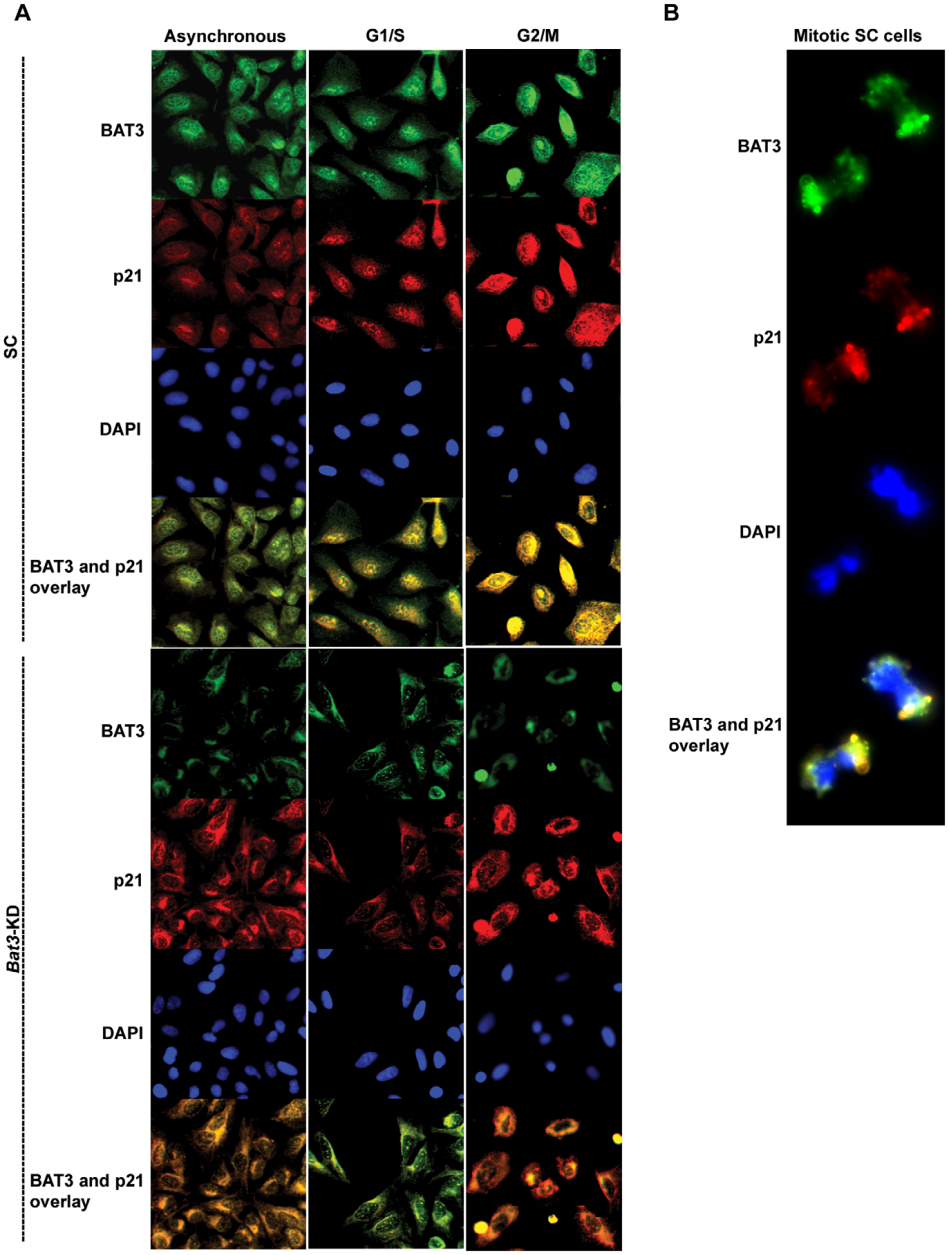
BAT3 co-localizes with p21 during the cell cycle. (A) Scrambled control (SC) and *Bat3*-knockdown (*Bat3*-KD) U2OS cells were synchronized at G1/S (middle column) and G2/M (right column), respectively. The cells were then fixed and stained for BAT3 (green) and p21 (red). (B) Mitotic SC cells were fixed and stained for BAT3 (green) and p21 (red).

Next, we determined whether BAT3 and p21 co-localize during the cell cycle. To do this, we synchronized the SC and *Bat3*-KD U2OS cells at G1/S and G2/M respectively, and performed immunofluorescence staining for BAT3 and p21 (Figure 8A). In the SC cells, BAT3 was observed to co-localize with p21. Moreover, both proteins were observed in the perinuclear region during G1/S (top panel, column 2), and in the nucleus during G2/M (top panel, columns 3, Table S1). On the other hand, in the *Bat3*-KD cells, BAT3 staining was detected only in the cytoplasm (bottom panel), in agreement with previous observations [4]. More importantly, in *Bat3*-KD cells, p21 remained cytoplasmic in both the asynchronous and synchronized populations. Staining of mitotic SC cells also revealed that BAT3 was localized to the spindle poles together with p21 (Figure 8B), providing further evidence that BAT3 plays a role in regulating mitosis (see Discussion).

However, we could not determine a direct interaction between endogenous BAT3 and p21 by co-immunoprecipitation (Figure S6C). These observations suggest that the interaction between BAT3 and p21 may be transient and/or indirect, and may also involve binding to other proteins. A recently-published automated yeast two-hybrid study identified BAT3 as a potential interacting partner of p21 and predicted BAT3 to be upstream of p21 [42], which is consistent with our observation that the two proteins co-localize in the U2OS cells. The yeast-two-hybrid study also predicted the interaction between p21 and BAT3 to be weak, thus it may be transient and/or indirect, consistent with our inability to detect any interaction by co-immunoprecipitation thus far. Nonetheless, the results of this yeast-two-hybrid study support our hypothesis that BAT3 is functionally linked to p21.

## Discussion

To date, BAT3 has been thought to function only in regulating apoptosis. We have, however, determined a novel, non-apoptotic role for BAT3 in promoting the switch between the pro- and anti-proliferative roles of p21 during the cell cycle. Specifically, BAT3 appears to regulate the activity, abundance and intracellular localization of p21 to facilitate G1/S transition and G2/M progression. In addition to defining a specific function for BAT3 in cell-cycle regulation, our findings also support the notion of a complex role for p21, both as an inhibitor and activator of cell-cycle progression [22], [23], [25], [31], [32], [33], [43]. Based on our findings, we propose that BAT3 promotes switching between the opposing activities of p21 by regulating p21 phosphorylation and synthesis, as well as the translocation of p21 between the cytoplasm and the nucleus.

### BAT3 Regulates p21 Phosphorylation and the Dynamic Equilibrium between its Synthesis and Degradation During the Cell Cycle

Based both on previous observations [23] and those that we have reported here, we propose that p21 phosphorylation during G2/M progression serves two purposes, namely to facilitate cyclin B/Cdk1 activation by p21, and to reduce the abundance of p21 protein in order to allow cells to progress through these cell-cycle phases. In this context, BAT3 promotes p21 phosphorylation, which in turn signals cells to enter mitosis. When mitosis is complete, the level of phosphorylated p21 declines. This is followed by a reduction in total p21 protein level, thus allowing the cells to enter the next cell cycle and begin p21 protein synthesis as needed.

It remains unclear what molecular event promotes the decline in phosphorylated p21 levels as cells exit mitosis. One possibility is that phosphorylated p21 is degraded; p21 phosphorylation by various kinases has been proposed to promote p21 degradation [20], [44], [45]. On the other hand, it is also possible that p21 dephosphorylation precedes its degradation as cells exit mitosis. Our findings favor the latter possibility because we observed that p21 was dephosphorylated in lysates generated from nocodazole-arrested cells when the phosphatase inhibitors were diluted below their effective concentrations. Moreover, we found that p21 accumulated rapidly in *Bat3*-KD cells released from a nocodazole arrest, whereas the phosphorylation of p21 was reduced. Degradation of p21 by APC/C^Cdc20^ during G2/M progression has also been reported to occur during prometaphase and the interaction between Cdk2 and p21 inhibits this process [17], [46]. These findings suggest that the decreased p21 phosphorylation in the *Bat3*-KD cells results in continuous p21 synthesis. Persistent p21 expression in *Bat3*-KD cells means that they are likely to persist for longer in G2 and are less likely to progress through mitosis into G1 of the next cell cycle. This explanation agrees with the findings of past studies showing that p21 protein abundance peaks in G2 [17], [25]. Hence, we propose that during G2/M progression, BAT3 facilitates p21 phosphorylation and its subsequent activity in promoting G2/M progression. As cells exit mitosis, p21 dephosphorylation serves both to signal its degradation as well as to halt its synthesis. Reduced BAT3 protein level in turn leads to decreased p21 phosphorylation and subsequently deprives the cells of this cue. As a result, the equilibrium between p21 synthesis and degradation is disrupted because the cells continue to synthesize p21 even when it is no longer required, and the reduced degradation of p21 results in consistently higher levels of p21 protein.

### The Implications of BAT3-mediated Translocation of p21 on its Function and Regulation During the Cell Cycle

The observed co-localization of BAT3 and p21 throughout the cell cycle prompted us to postulate that BAT3 mediates the translocation of p21 during the cell cycle as a scaffold protein. Although we could not observe a direct interaction between the two proteins via co-immunoprecipitation, one possibility is that BAT3, potentially acting in concert with other as yet unknown proteins, recruits p21 into a complex to promote its phosphorylation and translocation to the appropriate cellular compartments during the cell cycle. Several studies have indicated that intracellular translocation of p21 is crucial to p21 function. As mentioned earlier in this report, degradation of p21 at the G1/S border has been reported to occur solely in the nucleus, and is facilitated by a complex consisting of 14-3-3τ, p21, MDM2 and the C8 subunit of the 20 S proteasome [35], [36], [40], [41]. The localization of p21 to the cytoplasm has also been shown to promote cell proliferation, potentially by facilitating the assembly of cyclin D/Cdk4 complexes which are required during G1 [22], [47], [48], [49]. Moreover, p21 localization to the nucleus is required for its phosphorylation [23] and has also been proposed to facilitate G2/M progression [25]. Our findings concerning p21 localization not only agree with the conclusions of prior studies but also indicate that BAT3 is required for p21 translocation during the cell cycle. Reducing the abundance of BAT3 protein in cells leads to p21 remaining mostly in the cytoplasm as its localization to the nucleus is markedly reduced. Changes in the subcellular localization of p21 as a result of reduced BAT3 protein abundance subsequently leads to the deregulation of p21 function during the cell cycle.

A mitotic role for BAT3 was first proposed when it was identified as a Chap1-binding protein in a yeast-two-hybrid screen [50]. Dsk2, the budding yeast ortholog of Chap1, has been shown to be required for G2/M progression as it plays a role in spindle pole body assembly [51], [52]. In another study on the function of the hSGT protein during the cell cycle, it was observed that BAT3 forms a complex with hSGT and Hsp70 during prometaphase in HeLa cells [53]; findings from this study also showed that hSGT is localized to the spindle poles, although experimental limitations at the time did not allow the authors to determine the intracellular localization of BAT3. We have determined the intracellular localization of BAT3 during mitosis (Figure 8B) and confirmed that BAT3 has a mitotic function in U2OS cells.

The idea that the intracellular localization of BAT3 is important for its biological function is not surprising because a similar phenomenon has been observed for other mammalian BAG protein family members [1]. For instance, the subcellular localization of mammalian BAG-1, the most well-studied BAG protein, has been proposed as a good indicator of the prognoses for some cancers, although the outcome of nuclear and cytoplasmic localization of BAG-1 could be drastically different between different cancer types [54], [55], [56]. Furthermore, isoforms of BAG-1 have been shown to bind DNA and regulate transcription, indicating that nuclear localization of these isoforms is important for their function [57]. Subcellular localization of other mammalian BAG protein has also been shown to regulate a variety of biological functions, mostly via their role as co-chaperones with Hsp70 and Hsp90 [58], [59], [60]. More importantly, nuclear localization of BAT3 has been shown to be required to mediate p53-dependent apoptosis in response to DNA damage [4]. Interestingly, the subcellular localization of BAG proteins in plants has also been proposed to be important for their cellular function [1]. Although not much is currently known about the function of many of the BAG family proteins, their correct intracellular localization appears to be a universal requirement for their proper *in vivo* function.

### A Proposed Model for BAT3 Function During the Cell Cycle

**Figure 9:**
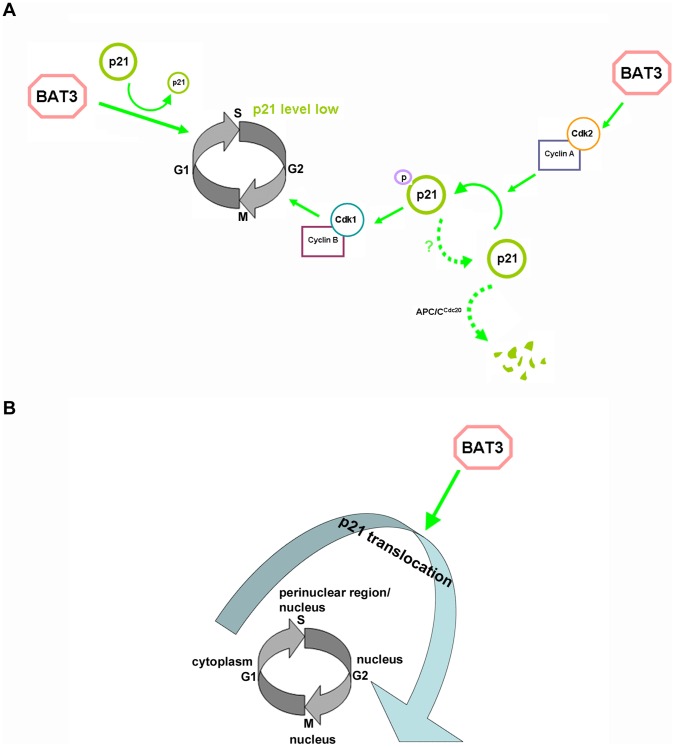
A model for BAT3 function during cell-cycle progression. (A) BAT3 facilitates both G1/S transition and G2/M progression by regulating p21 protein abundance and activity. (B) BAT3 facilitates the translocation of p21 between the cytoplasm and nucleus of the cells.

Based on our findings and available data in the literature [17], [23], [25], we propose the following model for how BAT3 regulates cell-cycle progression (Figure 9A). During the G1/S transition, BAT3 regulates p21 protein level and ultimately leads to a reduced abundance of p21 protein, which will then allow cells to proceed into S phase and undergo DNA replication. During G2/M progression, BAT3 promotes p21 phosphorylation by cyclin A/Cdk2, which in turn leads to the activation of cyclin B/Cdk1 and subsequent G2/M progression. BAT3 regulates Cdk2 kinase activity through cyclin A abundance. As the cells exit mitosis, p21 is degraded via an APC/C^cdc20^-dependent pathway.

Additionally, we postulate that p21 phosphorylation functions as the initiator of a chain of events resulting in G2/M progression, p21 dephosphorylation and its subsequent degradation. This is because although p21 degradation during G2/M has been shown to be ubiquitin-dependent [17], we were unable to detect ubiquitinated forms of p21 in our MG132 treatment experiment. Instead, we observed an accumulation of phosphorylated p21 (Figure 4B). These observations suggest that dephosphorylation of p21 is the pre-requisite for its ubiquitination. More importantly, the interaction between Cdk2 and p21 has been shown to inhibit the APC/C^Cdc20^-mediated degradation of p21 during G2/M progression [46]. These findings further supports our hypothesis that p21 is dephosphorylated as the cell exit mitosis. On the other hand, the reduction in BAT3 protein level results in the deregulation of p21 and ultimately leads to the accumulation of p21 protein. In other words, the fine balance of p21 protein level during G1/S transition and G2/M progression is perturbed and this subsequently leads to a delay in cell-cycle progression.

We propose that BAT3 promotes the translocation of p21 between the cytoplasm and the nucleus, thus facilitating the proper regulation of p21 function within the appropriate subcellular compartments during specific phases of the cell cycle (Figure 9B). Since we were unable to detect a direct interaction between BAT3 and p21, however, we believe that this interaction may be transient and/or indirect and might involve other proteins. One potential interactor is Cdk2, which has been proposed to phosphorylate p21 during G2/M progression [23]. Experiments aimed at identifying proteins that interact with BAT3 during the cell cycle may shed light on how it regulates p21.

Taken together, our study demonstrates for the first time that BAT3, a protein with a well-documented role in regulating apoptosis, is also important in the regulation of normal cell-cycle progression. Since many studies on BAT3 thus far have been focused on the role of BAT3 in regulating various stress-induced cellular responses [3], [4], [5], [61], [62], our findings highlight the fact that BAT3 regulates cellular responses under both stress-induced and normal conditions. In agreement with this conclusion, a recently-identified single nucleotide polymorphism (SNP) in BAT3 has been associated with high risk in non-small cell lung cancer [63], [64],[65]. These findings raise the exciting possibility that, apart from impairing apoptosis, this SNP might also lead to defects in cell-cycle progression. Detailed research in this area will help elucidate how BAT3 is able to function in regulating both apoptosis and normal cell-cycle progression under various cellular conditions.

## Materials and Methods

### Cell Culture

Stable, inducible U2OS cell lines previously generated in our lab [24] were cultured in McCoy’s 5A (Mediatech) supplemented with 10% FBS and penicillin/streptomycin at 37°C in 5% CO_2_ with the addition of 1.5 µg/ml puromycin (Sigma-Aldrich). For experiments using the U2OS stable cell lines, the cells were first treated with 2 µg/ml doxycycline (Dox, Sigma-Aldrich) in the presence of puromycin for 3 days. On day 4, cells were passaged and cultured overnight. On day 5, 2 µg/ml Dox was added to the cells (in the absence of puromycin). The cells were then used for experiments on day 6. Parental U2OS cells (ATCC) and HCT116 cells [66] were cultured in McCoy’s 5A and DMEM, respectively (both from Mediatech) supplemented with 10% FBS and penicillin/streptomycin. Transient transfection of siRNA targeting *Bat3* was performed using Lipofectamine 2000 (Invitrogen) following manufacturer’s instructions.

### Nocodazole Treatment

Cells were treated with either 100 or 800 ng/ml nocodazole (Sigma-Aldrich) for various amounts of time, depending on the experiment. Nocodazole treatment experiment using parental U2OS and HCT116 cells was performed as previously described [23].

### Cell Synchronization

For G1/S synchronization (double thymidine block), cells were treated with 2.5 nM thymidine (Sigma-Aldrich) for 18 h, released into full culture medium for 9 h, and treated with 2.5 nM thymidine for another 16 h. Finally, the cells were released and harvested at various time points. For synchronization in G2/M, cells were treated with 100 ng/ml nocodazole for 24 h and harvested at various time points following release.

### MG132 Treatment

Cells were synchronized using either a double thymidine block or nocodazole arrest/release as described earlier. The cells were subsequently released into media either with or without 20 µM MG132 (Enzo Life Sciences) and harvested at various time points. For the MG132 samples, MG132 was added to the cells 2 h before release.

### Cycloheximide (CHX) Treatment

Cells were synchronized using either a double thymidine block or nocodazole arrest/release as described earlier. The cells were subsequently released into medium with 40 µg/ml CHX (Sigma-Aldrich) and harvested at various time points. For cells synchronized by nocodazole arrest/release, only cells that were detached following nocodazole arrest were used for the release.

### Western Blot Analysis

Cells were lysed in universal lysis buffer (ULB) as described previously [67] with some modifications. For lysis of MG132-treated cells, 10 µg/µl each of aprotonin, leupeptin and pepstatin A, 2.5 mM sodium orthovanadate, 10 mM NEM (all from Sigma-Aldrich) and 25 µM MG132 were added to the lysis buffer. Alternatively, pellets of MG132-treated cells were boiled directly in SDS-PAGE sample buffer**.** All other cells were lysed similarly in ULB except that 1 µg/µl each of aprotonin, leupeptin and pepstatin A were used. Sodium molybdate was omitted in all lyses performed. Lysates were resolved on 15% low-bis SDS-PAGE gels with a 60∶1 mass ratio of acrylamide to bis-acrylamide. The following antibodies were used: rabbit anti-p21, rabbit anti-p53 (p-Ser15), mouse anti-cyclin A and mouse anti-actin (all from Cell Signaling Technology), mouse anti-actin (Santa Cruz Biotechnology), mouse anti-β-actin and chicken anti-BAT3 (both from Abcam).

### Flow Cytometry

Cells were fixed in chilled 70% ethanol and stored at −20°C. Fixed cells were washed with PBS and stained with propidium iodide. Flow cytometry was performed to analyze the DNA content of the cells using a FACScan analyzer. The DNA profiles were analyzed using the flow cytometry data analysis software, Cyflogic (CyFlo Ltd).

### Immunofluorescence for BAT3 and p21

Cells were plated on sterilized cover slips placed in a six-well plate and cultured overnight. Next, the cells were synchronized at either the G1/S boundary using double thymidine block or in G2/M using nocodazole, respectively. To prepare the cells for staining, the cells were washed with phosphate buffer saline (PBS) and fixed with a methanol:acetone solution at a volume ratio of 1∶1. The cells were then washed twice with PBS and re-hydrated with PBS for 5 min. The cells were incubated in 3% BSA in PBS for 1 h at room temperature. The primary antibody mix was prepared using 3% BSA in PBS and the cells were incubated with the primary antibodies overnight at 4°C. The cells were subsequently washed three times in PBS for 5 min each time and incubated with fluorophore-conjugated secondary antibodies for 1 h at room temperature. After three 5-min washes in PBS, the cells were mounted on slides with VectaShield mounting medium containing 4′,6′-diamidino-2-phenylindole (DAPI) (Vector Laboratories Inc.) and analyzed by fluorescence microscopy. The following antibodies were used: chicken anti-BAT3 (Abcam), rabbit anti-p21 (Cell Signaling Technology), FITC-tagged rabbit anti-chicken (Abcam) and Alexafluor 568 (Molecular Probes). Mitotic cells were obtained by collecting the cells that were detached following nocodazole arrest and staining was carried out as described above.

## Supporting Information

Figure S1 **Phosphatase treatment of lysates from G2/M-enriched scrambled control (SC) U2OS cells reduced the intensity of the slower-migrating p21 band.** The cells were treated with 100 ng/ml nocodazole for 24 h. Ppase, λ-phosphatase.
(TIF)

Figure S2 **p21 phosphorylation is not due to Dox treatment alone.** The scrambled control (SC) and *Bat3*-knockdown (B3) U2OS cells were treated with nocodazole (100 ng/ml) for 24 h in the presence and absence of Dox. Lysates from these cells were used for p21 western blot. Dox, Doxycycline.
(TIF)

Figure S3 **Caspase-3 cleavage is not detectable in lysates from nocodazole-treated scrambled control (SC) and** ***Bat3*** **-knockdown** **(** ***Bat3*** **-KD) cells.** Western blot for caspase-3 using lysates from SC and *Bat3*-KD U2OS stable cell lines treated with 100 ng/ml nocodazole for the various times indicated. Cytochrome C-treated Jurkat cell lysate was used as a positive control for caspase cleavage (Lane 1). C, positive control lysate.
(TIF)

Figure S4 **Analysis of p21 status following release from G1/S synchronization into medium containing nocodazole.** Western blot for p21 using lysates from the scrambled control (SC) and *Bat3*-knockdown (*Bat3*-KD) U2OS cells following release from G1/S synchronization into medium with 100 ng/ml nocodazole. A, asynchronous population.
(TIF)

Figure S5 **Analysis of the potential mechanism of BAT3-mediated p21 regulation during the cell cycle.** (A) Semi-quantitative RT-PCR of p21 mRNA in *Bat3*-knockdown (*Bat3*-KD) cells released from nocodazole arrest. (B) Western blot for phosphorylated Cdk2 at Tyr15 using lysates from mitotic scrambled control (SC) and *Bat3*-KD U2OS stable cell lines. The cells were treated with 100 ng/ml nocodazole for 24 h and the lysates were made from the cells that were detached following nocodazole treatment. A, asynchronous population; M, mitotic cells.
(TIF)

Figure S6 **Cell-cycle-dependent localization of BAT3.** (A) SaOS-2 cells were synchronized at G1/S and G2/M, respectively, fixed and stained for BAT3. (B) CCD-34Lu cells were harvested every 4 h following release from G1/S synchronization, fixed and stained for BAT3. (C) Western blot for BAT3 and p21 following immunoprecipitation. Lysates were generated from scrambled control (SC) U2OS cells treated with 100 ng/ml nocodazole for 24 h. Sup, supernatant; IP, immunoprecipitation.
(TIF)

Table S1 **Percentage of G2/M-arrested scrambled control (SC) and** ***Bat3*** **-knockdown (** ***Bat3*** **-KD) cells with nuclear co-localization of BAT3 and p21.** Values shown are the sum of cell counts from four independent experiments.
(DOC)

Information S1 **Supporting materials and methods are detailed in Information S1.**
(DOC)
