# Supplementary figures and images for: A Novel, Non-Apoptotic Role for Scythe/BAT3: A Functional Switch between the Pro- and Anti-Proliferative Roles of p21 during the Cell Cycle
Source: PLoS One. 2012 Jun 27;7(6):e38085. doi: 10.1371/journal.pone.0038085 (PMC3384656; doi:10.1371/journal.pone.0038085)

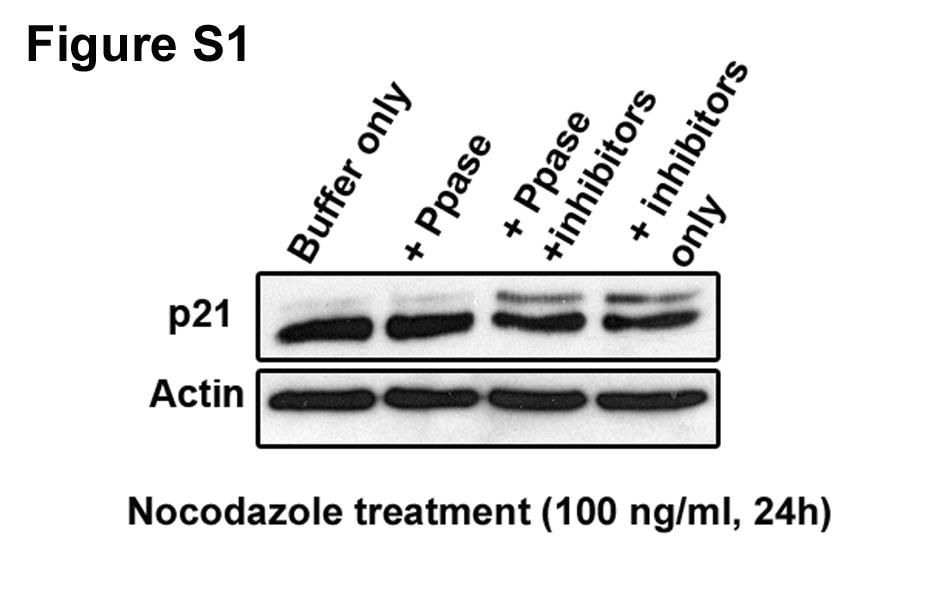

Supplement: Figure S1 — Phosphatase treatment of lysates from G2/M-enriched scrambled control (SC) U2OS cells reduced the intensity of the slower-migrating p21 band. The cells were treated with 100 ng/ml nocodazole for 24 h. Ppase, λ-phosphatase. (TIF) [file pone.0038085.s001.tif]

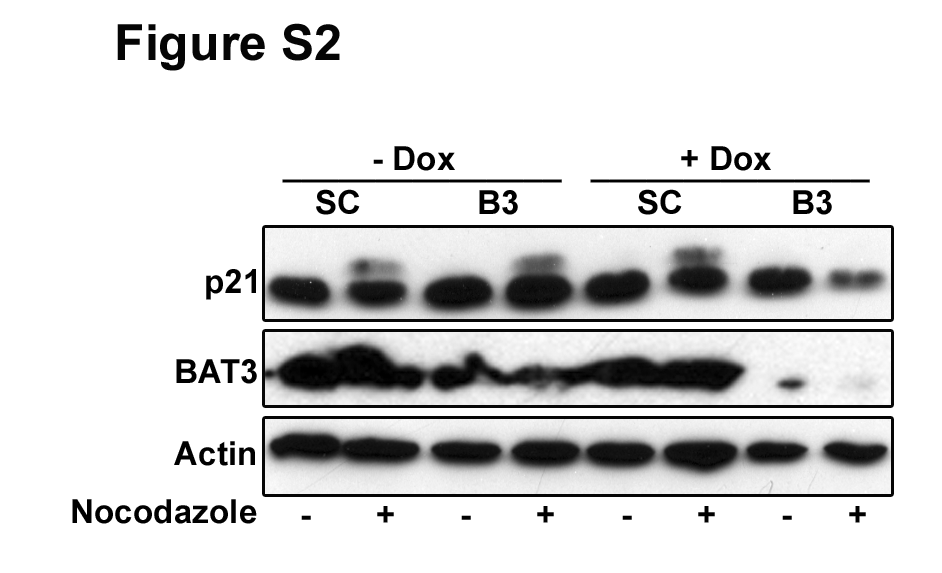

Supplement: Figure S2 — p21 phosphorylation is not due to Dox treatment alone. The scrambled control (SC) and Bat3-knockdown (B3) U2OS cells were treated with nocodazole (100 ng/ml) for 24 h in the presence and absence of Dox. Lysates from these cells were used for p21 western blot. Dox, Doxycycline. (TIF) [file pone.0038085.s002.tif]

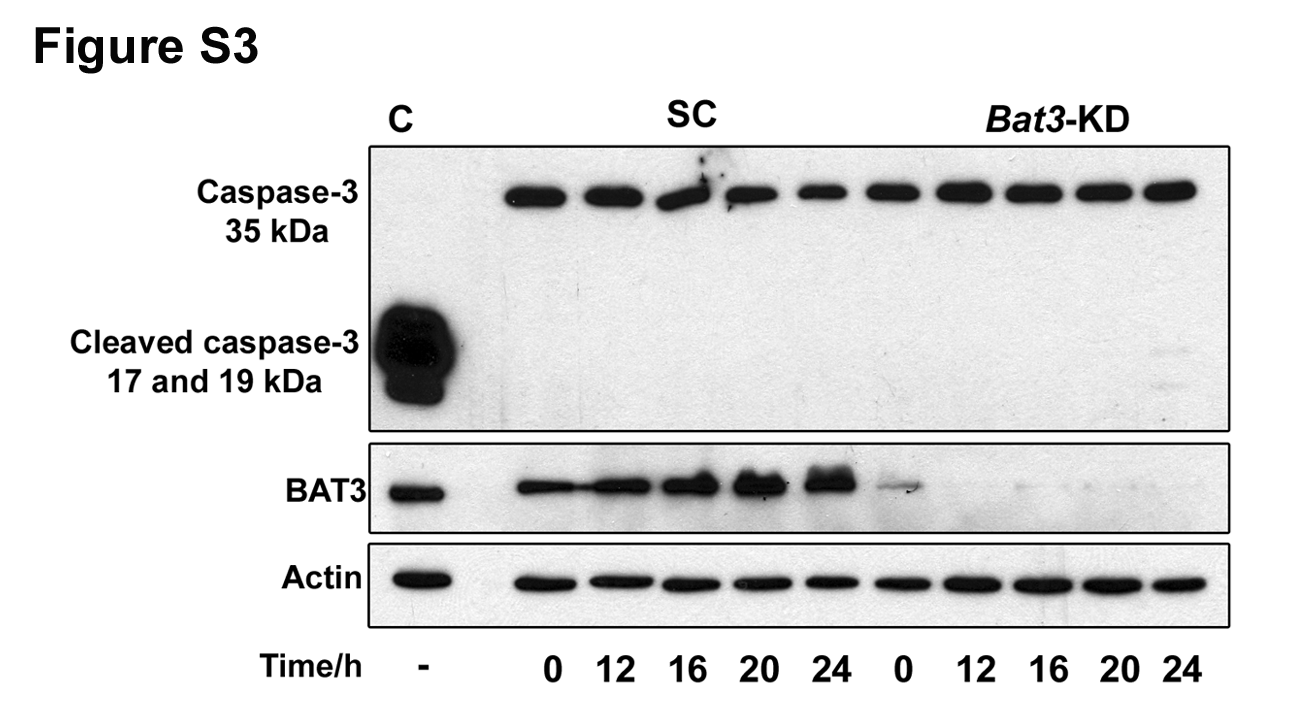

Supplement: Figure S3 — Caspase-3 cleavage is not detectable in lysates from nocodazole-treated scrambled control (SC) and Bat3 -knockdown ( Bat3 -KD) cells. Western blot for caspase-3 using lysates from SC and Bat3-KD U2OS stable cell lines treated with 100 ng/ml nocodazole for the various times indicated. Cytochrome C-treated Jurkat cell lysate was used as a positive control for caspase cleavage (Lane 1). C, positive control lysate. (TIF) [file pone.0038085.s003.tif]

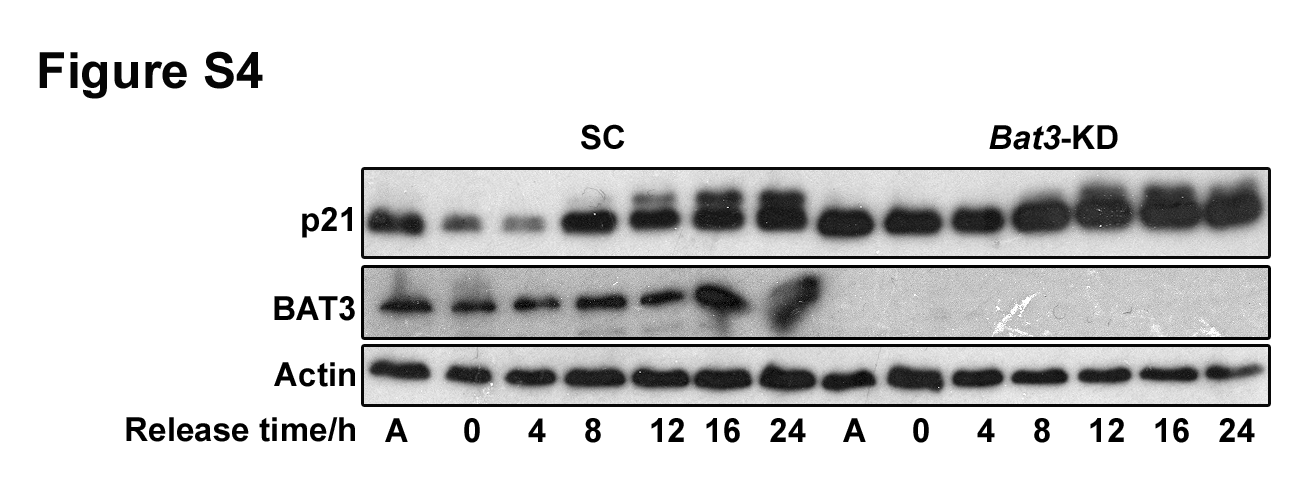

Supplement: Figure S4 — Analysis of p21 status following release from G1/S synchronization into medium containing nocodazole. Western blot for p21 using lysates from the scrambled control (SC) and Bat3-knockdown (Bat3-KD) U2OS cells following release from G1/S synchronization into medium with 100 ng/ml nocodazole. A, asynchronous population. (TIF) [file pone.0038085.s004.tif]

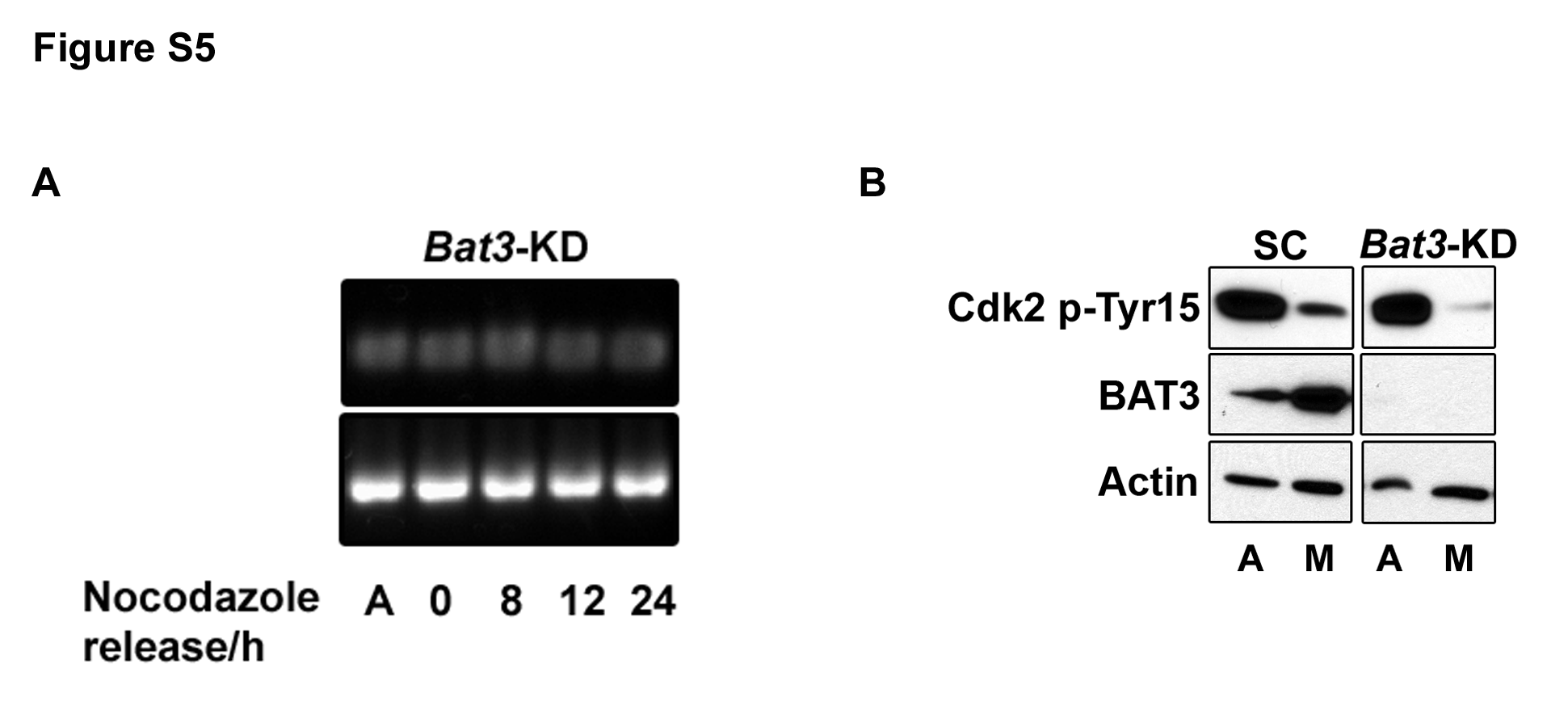

Supplement: Figure S5 — Analysis of the potential mechanism of BAT3-mediated p21 regulation during the cell cycle. (A) Semi-quantitative RT-PCR of p21 mRNA in Bat3-knockdown (Bat3-KD) cells released from nocodazole arrest. (B) Western blot for phosphorylated Cdk2 at Tyr15 using lysates from mitotic scrambled control (SC) and Bat3-KD U2OS stable cell lines. The cells were treated with 100 ng/ml nocodazole for 24 h and the lysates were made from the cells that were detached following nocodazole treatment. A, asynchronous population; M, mitotic cells. (TIF) [file pone.0038085.s005.tif]

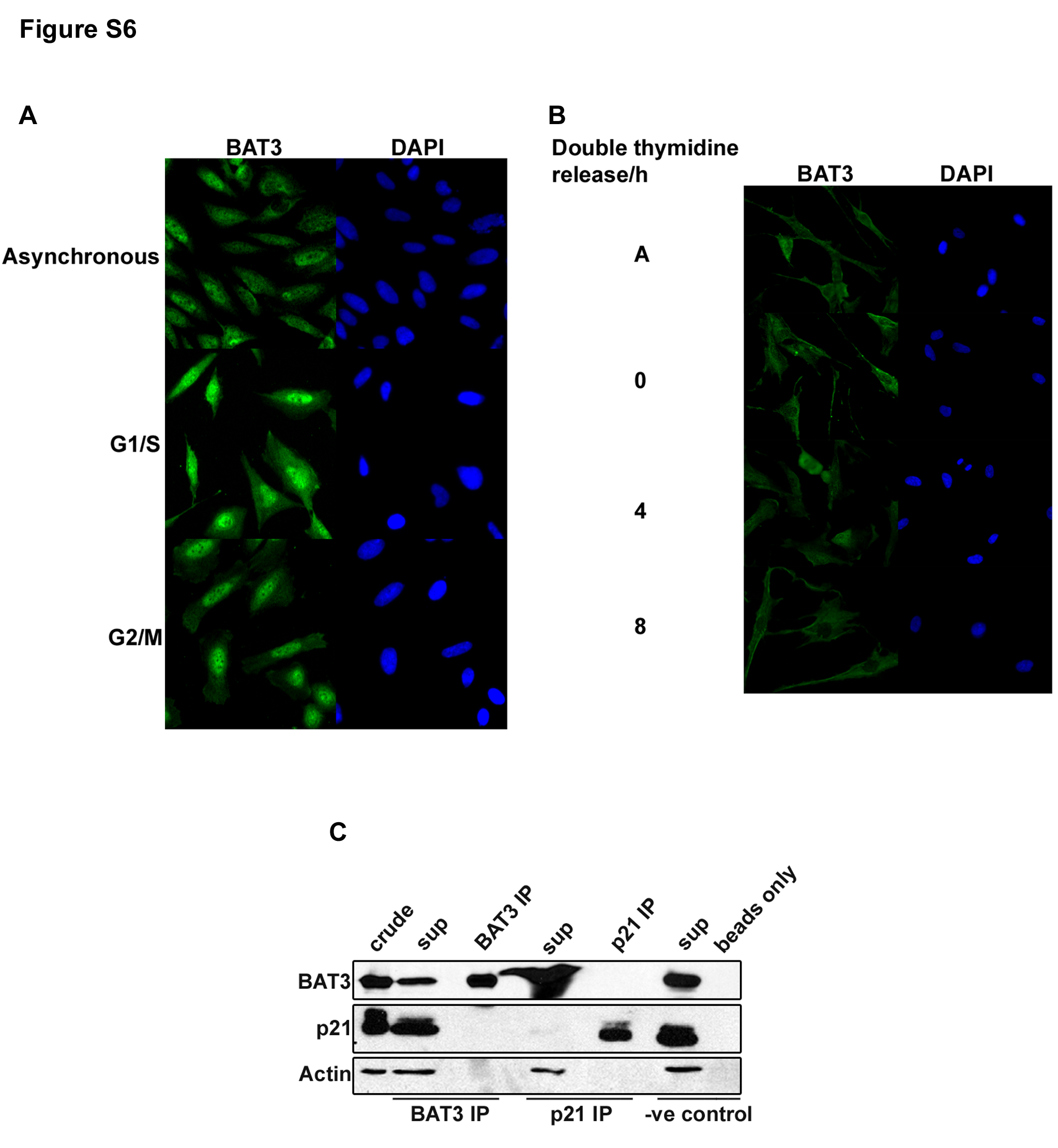

Supplement: Figure S6 — Cell-cycle-dependent localization of BAT3. (A) SaOS-2 cells were synchronized at G1/S and G2/M, respectively, fixed and stained for BAT3. (B) CCD-34Lu cells were harvested every 4 h following release from G1/S synchronization, fixed and stained for BAT3. (C) Western blot for BAT3 and p21 following immunoprecipitation. Lysates were generated from scrambled control (SC) U2OS cells treated with 100 ng/ml nocodazole for 24 h. Sup, supernatant; IP, immunoprecipitation. (TIF) [file pone.0038085.s006.tif]
