# Supplementary material for: A Novel, Non-Apoptotic Role for Scythe/BAT3: A Functional Switch between the Pro- and Anti-Proliferative Roles of p21 during the Cell Cycle
Source: PLoS One. 2012 Jun 27;7(6):e38085. doi: 10.1371/journal.pone.0038085 (PMC3384656; doi:10.1371/journal.pone.0038085)
Supplement: Table S1 — Percentage of G2/M-arrested scrambled control (SC) and Bat3 -knockdown ( Bat3 -KD) cells with nuclear co-localization of BAT3 and p21. Values shown are the sum of cell counts from four independent experiments. (DOC) [file pone.0038085.s007.doc]

### Table S1

| **Cell line** | **Percentage** |
| --- | --- |
| SC | 82.3 |
| *Bat3*-KD | 13.5 |
