# Supplementary material for: A Novel, Non-Apoptotic Role for Scythe/BAT3: A Functional Switch between the Pro- and Anti-Proliferative Roles of p21 during the Cell Cycle
Source: PLoS One. 2012 Jun 27;7(6):e38085. doi: 10.1371/journal.pone.0038085 (PMC3384656; doi:10.1371/journal.pone.0038085)
Supplement: Information S1 — Supporting materials and methods are detailed in Information S1. (DOC) [file pone.0038085.s008.doc]

## Information S1

### Supporting Materials & Methods

*SaOS-2 and CCD-34Lu cell culture -* SaOS-2 and CCD-34Lu cells (both from ATCC) were cultured in McCoy’s 5A and Minimum Essential Medium, respectively (both from Mediatech), supplemented with 10% FBS and penicillin/streptomycin at 37ºC in 5% CO_2_. Synchronization was performed as described in Materials and Methods. 400 ng/ml of nocodazole was used for the G2/M synchronization of SaOS-2 cells.

*In vitro* *dephosphorylation assay for p21 -* Lysates from cells treated with nocodazole for 24 h were incubated either with or without λ-phosphatase (New England Biolabs) in a phosphatase assay reaction mix (x NEB PMP buffer, 1 mM MnCl­­_2_ and protease inhibitors) for 1 h at 30 °C. Other controls included lysates with both λ-phosphatase (200 U) and phosphatase inhibitors (50 mM NaF, 10 mM Na_3_VO_4_) added, and lysates with only phosphatase inhibitors added. The protease inhibitors used are the same as the ones used for cell lysis. The reactions were halted by boiling in SDS-PAGE sample buffer, then resolved on 15% low-bis SDS-PAGE gels (60:1 mass ratio of acrylamide to bis-acrylamide) and subject to western blotting for p21.

*Immunoprecipitation of BAT3 and p21 -*  Lysates from cells treated with nocodazole for 24 h were diluted in IPP150 (buffer composition as described in [1] except with 25 mM Tris-HCl) with phophatase and protease inhibitors added and incubated with either rabbit anti-BAT3 (gift from Sally Kornbluth, Duke University) or rabbit anti-p21 (Cell Signaling Technology) for 2 h at 4°C, after which the protein A agarose beads were added and the incubation resumed at 4°C overnight. The beads were subsequently washed three times with the immunoprecipitation buffer, boiled in SDS sample buffer and resolved on 15% low-bis SDS-PAGE gels (60:1 ratio of acrylamide to bis-acrylamide).

*Semi-quantitative RT-PCR for p21 –* Total RNA was isolated from *Bat3*-KD cells using the RNAqueous-4PCR kit (Life Technologies) following manufacturer’s instructions. cDNA was generated using Superscript III First Strand Synthesis System for RT-PCR (Invitrogen) and was used in subsequent PCR for p21 and β-actin. The following primers were used: 5'-CTGGGGATGTCCGTCAGAACCCATGC-3’ (p21 forward), 5'-GAGTCTCCAGGTCCACCTGGGGAC-3’ (p21 reverse) (modified from [2]); 5'-ATCTGGCACCACACCTTCTACAATGAGCTGCG-3’ (β-actin forward) and 5'-CGTCATACTCCTGCTTGCTGATCCACATCTGC-3’ (β-actin reverse) (previously published in [3]).

*Western blot for caspase-3 and phopho-Cdk2 (Tyr15) –* Lysates were prepared and western blotting was performed as described in the main text, except that SDS-PAGE for the caspase-3 blots were performed using 37.5:1 mass ratio acrylamide/bis-acrylamide gels. The cytochrome C-treated Jurkat cell lysate was purchased from Cell Signaling Technology. The following antibodies were used: rabbit anti-caspase-3 (Cell Signaling Technology) and rabbit anti-Cdk2 (p-Tyr15) (Abcam).

### References

1. Puig O, Caspary F, Rigaut G, Rutz B, Bouveret E, et al. (2001) The tandem affinity purification (TAP) method: a general procedure of protein complex purification. Methods 24: 218-229.

2. Sasaki T, Gan EC, Wakeham A, Kornbluth S, Mak TW, et al. (2007) HLA-B-associated transcript 3 (Bat3)/Scythe is essential for p300-mediated acetylation of p53. Genes Dev 21: 848-861.

3. Dong YG, Chen DD, He JG, Guan YY (2004) Effects of 15-deoxy-delta12,14-prostaglandin J2 on cell proliferation and apoptosis in ECV304 endothelial cells. Acta Pharmacol Sin 25: 47-53.
